# Electro‐Acupuncture to Treat Disorder of Consciousness (AcuDoc): Study Protocol for a Randomized Sham‐Controlled Trial

**DOI:** 10.1002/brb3.70637

**Published:** 2025-06-26

**Authors:** Kaiqi Lin, Jixiang Chen, Jiahui Pan, Ruihong Wang, Shibiao Wu, Wanxing Wen, Yuanqing Li, Lixin Wang, Fang Yuan

**Affiliations:** ^1^ The Second Clinical Medical College of Guangzhou University of Chinese Medicine Guangzhou China; ^2^ Department of Rehabilitation Medicine Guangzhou First People's Hospital Guangzhou China; ^3^ School of Software South China Normal University Guangzhou China; ^4^ Department of Neurocritical Care The Second Affiliated Hospital of Guangzhou University of Chinese Medicine Guangzhou China; ^5^ Center for Brain‐Computer Interfaces and Brain Information Processing South China University of Technology Guangzhou China; ^6^ State Key Laboratory of Traditional Chinese Medicine Syndrome/Department of Neurocritical Care The Second Affiliated Hospital of Guangzhou University of Chinese Medicine Guangdong Provincial Academy of Chinese Medical Sciences Guangzhou China

**Keywords:** disorder of consciousness, electro‐acupuncture, neuroimaging, randomized controlled trial, traumatic brain injury

## Abstract

**Background:**

Treatment of disorders of consciousness (DOC) remains a clinical challenge. Electroacupuncture (EA) was shown to have the potential to promote the recovery of consciousness. This trial aims to explore the therapeutic effects and mechanisms of EA in patients with DOC due to traumatic brain injury (TBI) through a multimodal approach.

**Methods:**

A total of 50 adult patients with DOC due to TBI and 25 healthy subjects will be enrolled in the study. Patients enrolled in the study will be assigned to the EA group or the sham‐EA group through stratified randomization. All patients receive behavioral assessments (CRS‐R and brain–computer interface), neurophysiological evaluations (EEG, somatosensory evoked potentials, brainstem auditory evoked potentials), and neuroimaging evaluations (rs‐fMRI, amide proton transfer, intravoxel incoherent motion, neurite orientation dispersion and density imaging) before and after the 14‐day EA or sham‐EA treatment. Each healthy subject will receive a set of neurophysiological and neuroimaging examinations but no treatments. The practitioner administering EA and sham‐EA is the only one aware of the grouping results. In the sham‐EA group, sham‐acupoints, sham‐acupuncture, and sham‐wire are utilized. The primary outcome measurement is the change in CRS‐R score after 14 days of treatment compared with the baseline CRS‐R score.

**Discussion:**

The AcuDoc trial will be the first randomized sham‐controlled study to investigate the clinical benefits of EA in patients with DOC. This trial will elucidate the role of EA in the treatment of DOC due to TBI and provide evidence of its therapeutic mechanisms.

## Graphical abstract

This randomized sham‐controlled trial aims to explore the therapeutic effects and mechanisms of electroacupuncture in patients with disorder of consciousness due to traumatic brain injury. It includes 50 patients and 25 healthy subjects. The primary outcome is the change in the CRS‐R score after 14 days of treatment.

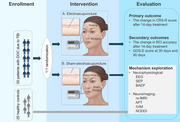

## 1 Background

With the rapid development of life support technology, an increasing number of patients can survive severe brain injury. Some survivors regain consciousness after a period of coma; however, many patients develop prolonged disorders of consciousness (DOC), which poses a therapeutic challenge for clinicians and a heavy burden for their families (Thibaut et al. 2019).

In recent years, many new therapeutic interventions have been attempted to improve the recovery of consciousness: pharmacological treatments include dopamine agonists and N‐methyl D‐aspartate antagonists (amantadine), gamma‐aminobutyric acid agonists (baclofen, zolpidem, and midazolam), and calcium channel blocker (ziconotide); non‐pharmacological treatments include transcranial direct current stimulation (targeting the cortex), repeated transcranial magnetic stimulation (targeting the cortex), low‐intensity focused ultrasound pulse (targeting the central thalamus), deep brain stimulation (targeting the central thalamus), vagal nerve stimulation (targeting the brainstem), spinal cord stimulation (targeting the brainstem), and sensory stimulations (targeting various sensory pathways) (Thibaut et al. 2019). However, only two trials have shown class II evidence (Giacino et al. 2012; Thibaut et al. 2014), and interventions stimulating other neural pathways and circuits also need to be explored.

According to traditional Chinese medicine theory, acupuncture at Shuigou point (also known as Renzhong, GV 26, DU 26) can “wake up the brain and open orifices” and has been used to improve the recovery of consciousness for more than a thousand years (Li and Liang 2016). In recent years, many studies have investigated the therapeutic effects and mechanisms of acupuncture at Shuigou in restoring consciousness. A meta‐analysis of 14 randomized controlled trials, with sample sizes ranging from 29 to 150, demonstrated that EA significantly improved the Glasgow Coma Scale and Glasgow Outcome Scale scores in traumatic brain injury (TBI) patients with coma, and Shuigou was the most commonly used acupoint in these trials (Chen et al. 2021). A self‐controlled functional near‐infrared spectroscopy study involving 16 DOC patients demonstrated that acupuncture at Shuigou increased the concentration of oxygenated hemoglobin in the prefrontal cortex and strengthened the connection strength of the left cerebral cortex (Xin et al. 2022). A self‐controlled motor‐evoked potential test study involving 14 DOC patients indicated that acupuncture at Shuigou increased the excitability of the cortico‐spinal system in patients with DOC following TBI (Matsumoto‐Miyazaki et al. 2016). Orexin is believed to stimulate cortical activation and regulate arousal, and acupuncture at Shuigou has been reported to promote the recovery of consciousness in TBI‐induced comatose rats via the upregulation of the expression of orexin‐1 and orexin‐1 receptor in the medial prefrontal cortex (Tan et al. 2021). Informed by traditional Chinese medical theory, ancient medical records, modern clinical practice, and relevant mechanistic studies, Shuigou has been selected as the primary acupoint in this study to facilitate the recovery of consciousness. However, it should be noted that the clinical trials included in the aforementioned meta‐analysis had several limitations. Specifically, these trials encompassed patients with both coma and prolonged DOC, utilized the Glasgow Coma Scale, which is not suitable for assessing prolonged DOC, to evaluate the severity and outcomes, and lacked sham controls. Although several studies have attempted to investigate the mechanism of acupuncture in treating DOC, the perspectives of these studies are rather singular and limited. A more in‐depth and multimodal investigation is required to evaluate the effects of acupuncture on the structure, perfusion, metabolism, electrophysiological activity, and function of critical areas in the consciousness circuitry.

Given the previous findings on the effects of EA on DOC, we initiated a randomized controlled trial called AcuDoc, dedicated to examining the efficacy of EA against DOC through behavioral and brain–computer interface (BCI) assessments and exploring its potential therapeutic mechanisms via neurophysiological and neuroimaging techniques.

## 2 Methods

### 2.1 Study Objectives

This study aims to explore the therapeutic effects and mechanisms of EA in patients with DOC due to TBI through a multimodal approach.

### 2.2 Study Design

**FIGURE 1:**
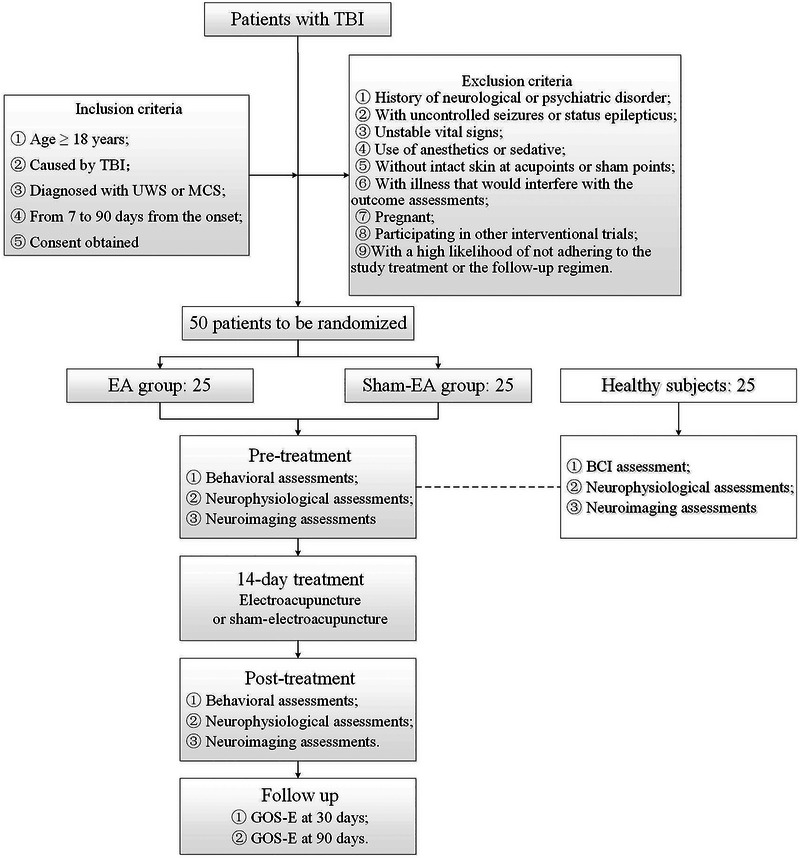
Study flow. BCI, brain–computer interface; EA, electroacupuncture; GOS‐E, Glasgow Outcome Scale‐Extended; MCS, minimally conscious state; TBI, traumatic brain injury; UWS, unresponsive wakefulness syndrome.

This is a single‐center, participant‐blinded, randomized, sham‐EA‐controlled trial. The AcuDoc trial will enroll 50 patients with DOC and 25 healthy subjects (Figure 1). Patients will be randomized to the EA group or the sham‐EA group. Each healthy subject will receive a set of neurophysiological and neuroimaging examinations but no treatments.

### 2.3 Ethics

The study is performed in accordance with the principles of the Declaration of Helsinki, has been approved by the ethics committee of the Second Affiliated Hospital of Guangzhou University of Chinese Medicine (BF2023‐252‐01), and has been registered at ClinicalTrials.gov (NCT06268236, on February 12, 2024). The protocol of this trial was performed according to the SPIRIT 2013 Statement (Supporting Information). Written informed consent will be obtained from the legal representative of each participant because DOC patients are not competent to provide informed consent.

Protocol amendments will be agreed upon with the AcuDoc Study Group, Sponsor, and Funding Body before submission for ethical approval. Following ethical approval, protocol modifications will be communicated with relevant parties such as the trial investigators and the trial registry.

### 2.4 Study Population

The study will include 50 adult patients with DOC due to TBI and 25 healthy subjects. Patients eligible for inclusion must meet the following criteria: (1) age ≥ 18 years; (2) with cerebral damage due to TBI; (3) diagnosed with unresponsive wakefulness syndrome (UWS) or minimally conscious state (MCS) based on at least two Coma Recovery Scale‐Revised (CRS‐R) assessments; (4) from 7 to 90 days from the onset of brain injury; (5) informed consent obtained.

Exclusion criteria are (1) with a history of neurological or psychiatric disorder before the brain injury; (2) with uncontrolled seizures or status epilepticus; (3) with unstable vital signs and requiring the use of vasoactive agents; (4) with the use of general anesthetics or central acting sedative; (5) without intact skin at acupoints or sham points; (6) with concomitant medical illness that would interfere with the outcome assessments and/or follow‐up; (7) pregnant patients; (8) currently participating in other investigational trials; (9) with high likelihood of not adhering to the study treatment or the follow‐up regimen.

Age‐ and sex‐matched healthy subjects will be included in this trial and must meet the following criteria: (1) without a history of brain injury; (2) without alcohol/substance abuse; (3) without neurological or psychiatric disorders; (4) currently not on any medication.

### 2.5 Randomization

As soon as informed consent is provided, the investigator should acquire the result of randomization. Stratified randomization, employing two strata (MCS and UWS), is utilized to ensure balanced proportions of UWS and MCS patients in each group. A secure randomization software is used to perform the randomization (computerized random numbers). Patients will be randomized in a 1:1 ratio to receive EA or sham‐EA treatment.

### 2.6 Masking

The EA and sham‐EA practitioners are the only investigators informed of the grouping results. Patients, their families, and all the other investigators are blinded to the allocation. To help maximize the blinding of participants, sham‐acupoints, sham‐acupuncture, and sham‐wire are used. A curtain is placed around the patient during the procedure, from the needle insertion to the determination of the maximum withstand current, to prevent other individuals from observing any physical reactions. Outcome scores are assessed independently by a qualified researcher who does not participate in the treatment and is blind to the grouping results.

### 2.7 Trial Interventions

All patients receive routine standardized neurocritical care management, including airway management, temperature regulation, blood pressure control, electrolyte balance maintenance, infection control, nutritional support, etc. Additionally, all patients undergo passive range of motion therapy and ergometer cycling therapy daily, administered by a physiatrist blinded to the group allocation.

**FIGURE 2:**
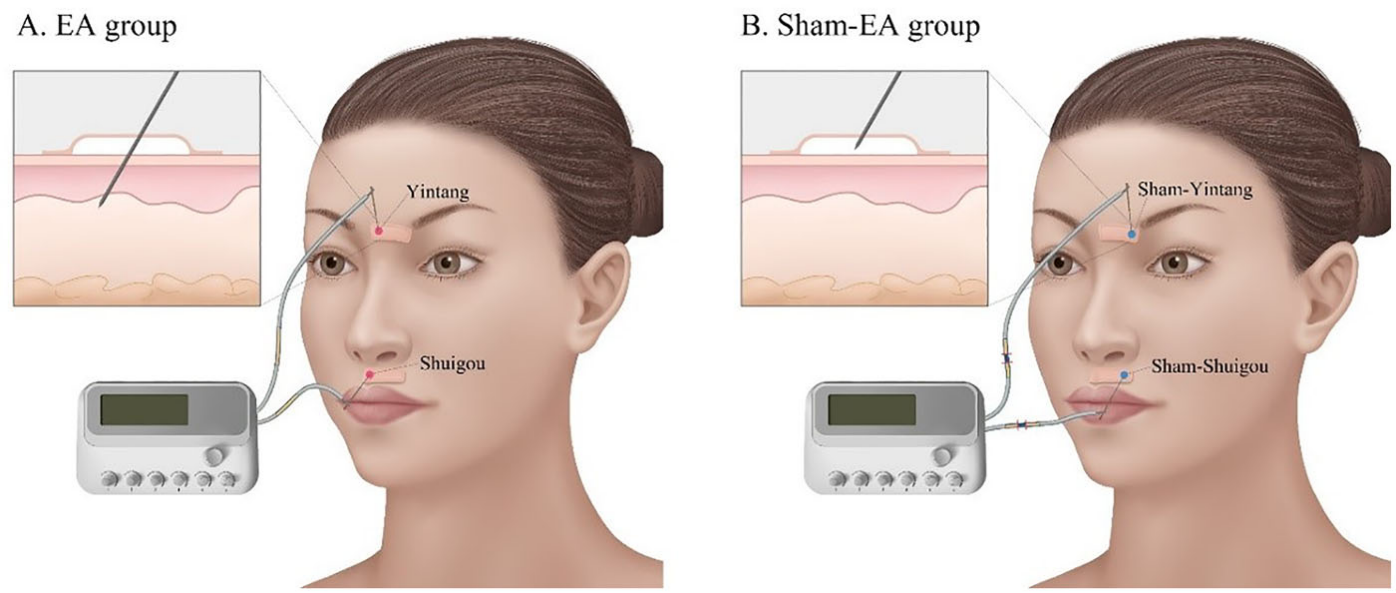
Electroacupuncture and sham‐electroacupuncture treatments. EA, electroacupuncture.

All patients receive EA or sham‐EA treatment in the supine position at 9 am for 30 min once a day for 14 consecutive days (Figure 2). One certified acupuncturist performs EA and sham‐EA treatments for patients in both groups. Disposable sterile acupuncture needles (0.25×25 mm, Hanyi brand, Changchun AIK Medical Devices Co., China) and EA apparatuses (SDZ‐V, Hwato Brand, Suzhou Medical Appliance Factory, China) are used in both groups.

Patients in the EA group receive acupuncture at Shuigou (also known as Renzhong, GV26, DU 26) and Yintang (updated number GV24+, previous number GV29). Shuigou is located on the midline above the upper lip, at the junction of the upper one‐third and lower two‐thirds of the philtrum midline (Institute of Acupuncture and Moxibustion, China Academy of Chinese Medical Sciences 2021). Yintang is located on the forehead, at the midpoint between the two medial ends of the eyebrow (Institute of Acupuncture and Moxibustion, China Academy of Chinese Medical Sciences 2021). After the skin is sterilized, sterile adhesive pads are placed in the area between the nose and upper lip and in the area between the eyebrows. Acupuncture needles are inserted through the adhesive pads approximately 10–15 mm into the skin. The acupuncture needle is inserted obliquely (30°–45°) upward to the direction of the nose for Shuigou and is inserted obliquely (15°–30°) downward to the direction of the nose for Yintang. Paired electrodes from the EA apparatus are clipped to the needle handles at Shuigou and Yintang. The EA stimulation lasts for 30 min with a wave of rarefaction and condensation (4 Hz/20 Hz) and a current intensity of the maximum withstand current within 10 mA. The maximum withstand current is defined as the maximum current intensity that does not cause any discomfort with a Nociception Coma Scale‐Revised score (Chatelle et al. 2012) above 4 and is different from the baseline value.

Patients in the sham‐EA group receive sham‐EA at sham‐Shuigou (1 cun [≈20 mm] lateral to Shuigou) and sham‐Yintang (1 cun [≈20 mm] lateral to Yintang). After the skin is sterilized, sterile adhesive pads are placed in the area between the nose and upper lip and in the area between the eyebrows. Acupuncture needles are inserted into the adhesive pads but do not pierce the skin. Paired electrodes from the EA apparatus via sham connecting cords are clipped to the needle handles at sham‐Shuigou and sham‐Yintang. The sham connecting cords are similar in appearance to the normal ones, but the inner wires in the sham connecting cords are cut off and cannot conduct electricity. The sham‐EA stimulation lasts for 30 min with a wave of rarefaction and condensation (4 Hz/20 Hz) and a current intensity of the maximum withstand current within 10 mA.

### 2.8 Study Procedures

At the time point of screening, demographics, education, time from injury, injury location, right‐ or left‐handed, medical history, current medications, CRS‐R score, and state of DOC are recorded (Table 1). After the enrollment, the following neurophysiological and neuroimaging examinations are performed before the first EA or sham‐EA treatment: EEG, somatosensory evoked potential (SEP), brainstem auditory evoked potential (BEAP), resting‐state functional MRI (rs‐fMRI), amide proton transfer (APT) imaging, intravoxel incoherent motion (IVIM) imaging, neurite orientation dispersion and density imaging (NODDI), and BCI. During the 14 days of treatment, the CRS‐R score is assessed every day after the treatment. After the last EA or sham‐EA treatment, the CRS‐R score and the above‐mentioned neurophysiological and neuroimaging examinations are performed again. During hospitalization, vital signs and adverse events (AEs) are monitored, and concomitant treatments are documented. Glasgow Outcome Scale‐Extended (GOS‐E) score at 30 and 90 days after randomization are collected.

**TABLE 1:** Timing and content of the study assessments.

Items	Day of enrollment					
	Screening	Pre‐treatment assessments	14‐day treatment	Post‐treatment assessments	Follow up	
					30 days	90 days
Written informed consent	●					
Inclusion and exclusion criteria	●					
Demographics	●					
Education	●					
Time from injury	●					
Brain injury location	●					
Right‐ or left‐handed	●					
Medical history	●					
Current medication	●					
Physical examination	●					
CRS‐R score	●		●	●		
State of consciousness	●		●	●		
Brain–computer interface assessment		●		●		
EEG, SEP, BAEP		●		●		
rs‐fMRI, APT, IVIM, NODDI		●		●		
GOS‐E					●	●
Vital signs monitoring	●	●	●	●		
Adverse events			●	●		
Concomitant therapies	●					

Abbreviations: APT, amide proton transfer; BAEP, brainstem auditory evoked potential; CRS‐R, Coma Recovery Scale‐Revised; EEG, electroencephalogram; GOS‐E, Glasgow Outcome Scale‐Extended; IVIM, intravoxel incoherent motion; NODDI, neurite orientation dispersion and density imaging; rs‐fMRI, resting‐state functional MRI; SEP, somatosensory evoked potential.

### 2.9 Assessment of the Level of Consciousness

**FIGURE 3:**
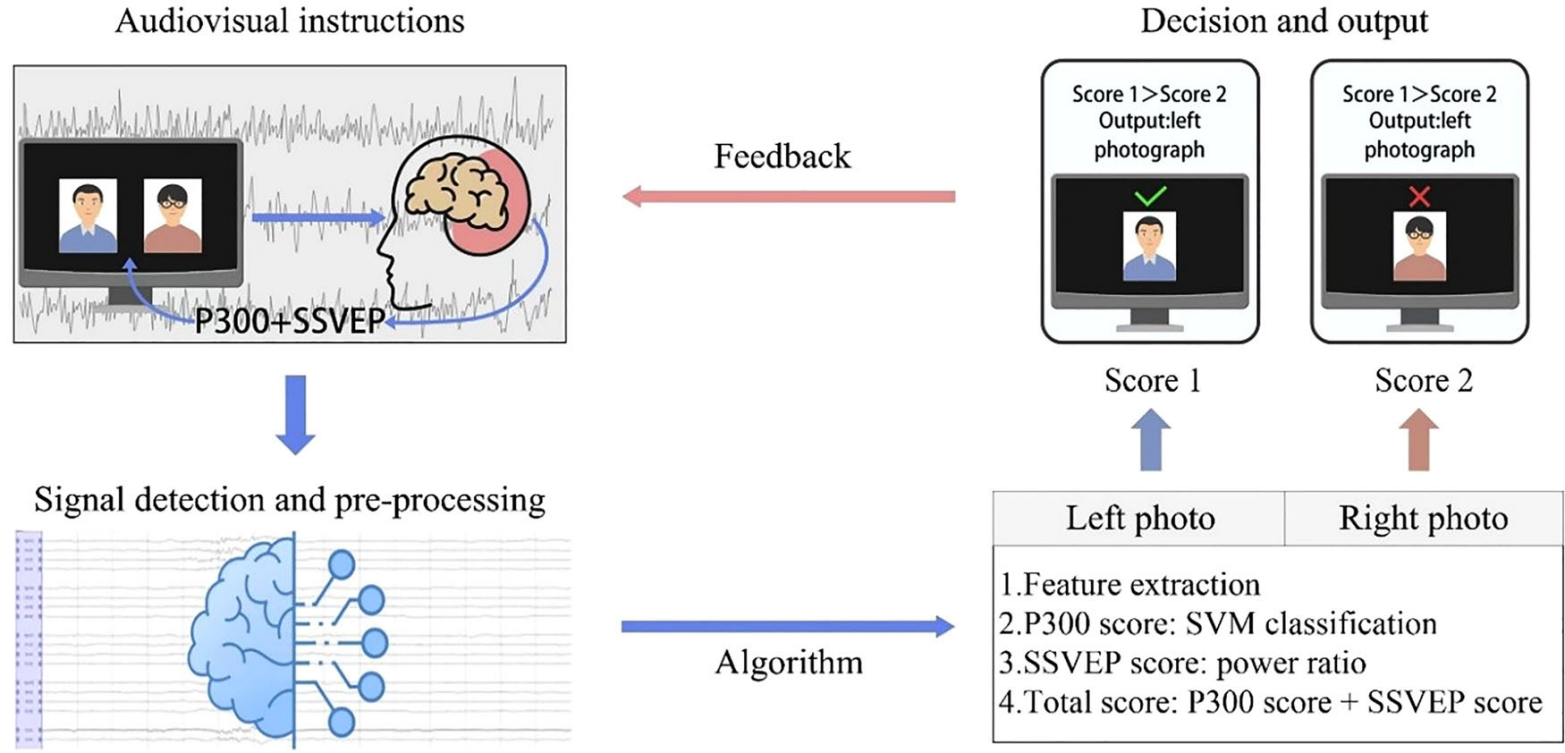
Brain–computer interface assessment system. SSVEP, steady‐state visually evoked potential; SVM, support vector machine.

The level of consciousness is assessed using CRS‐R scores and a BCI system (Figure 3), which is conducted by trained researchers who are blinded to the grouping results. The CRS‐R has a maximum score of 23 points (higher scores indicate higher levels of consciousness) and consists of 23 items broken into 6 subscales: auditory, visual, motor, verbal, communication, and arousal functions. The Chinese version of CRS‐R is used in this study (Di et al. 2017; Zhang et al. 2019). All patients receive three CRS‐R assessments administered at 24‐h intervals​​ during both pre‐treatment screening and post‐treatment evaluation. Diagnoses of UWS, MCS (including MCS‐ and MCS+), and emergence from the minimally conscious state (EMCS) are based on at least two CRS‐R assessments (Bruno et al. 2011; Giacino et al. 2002), with the higher score designated as the baseline and outcome CRS‐R score for each patient. Additionally, daily CRS‐R assessments are conducted throughout the 14‐day treatment period.

Our previously published EEG‐based BCI system is also used to assess the level of consciousness by analyzing P300 and steady‐state visual evoked potentials (SSVEP) when participants perform cognitive tasks (Pan et al. 2020). Briefly, participants are asked to focus on a photo of themselves or a randomly selected stranger and count the flashes of the photo's frame according to audiovisual instructions. The photograph‐related P300 and SSVEP are detected and analyzed. A total of 50 trials are given to each patient. BCI accuracy is calculated as the ratio of the number of trial successes to the total number of trials and is considered a parameter related to the level of consciousness.

### 2.10 Neurophysiological Assessments

All patients receive two times of video‐EEG monitoring. The first EEG recording starts 20 min before the first EA or sham‐EA treatment and continues until 24 h after the treatment. The second EEG recording starts 20 min before the last EA or sham‐EA treatment and continues until 24 h after the treatment. EEG signals are collected through 19‐channel monopolar leads with Ag/AgCl cup electrodes (NicoletOne EEG System, USA), placed according to the international 10–20 system. The sampling frequency is 500 Hz and the electrode–skin impedance is lower than 5 kΩ.

All patients receive SEP and BAEP assessments before and after the 14‐day EA or sham‐EA treatment (Nicolet Monitor UltraPro S100, US). All SEPs and BAEPs are assessed for reproducibility and analyzed by one certified neurophysiologist. For SEP, monophasic rectangular‐wave 200 µs stimulus pulses are given to the median nerve, and the intensity is adjusted to produce a visible thumb twitch. A minimum of 500 stimulations are averaged per recording. For BAEP, monoaural click stimuli of 50 µs duration are delivered through shielded headphones at a frequency of 10 Hz and an intensity of 70–90 dB HL. Both ears are stimulated separately. The contralateral ear is masked by 20 dB noise. Three trials of 1,000 clicks are recorded to assess reproducibility.

### 2.11 Neuroimaging Assessments

All patients receive two sets of neuroimaging examinations: one is before EA or sham‐EA treatment, and the other is after all the treatments. One set of neuroimaging examinations includes rs‐fMRI, APT, IVIM, and NODDI. MRI data are acquired using a 3T magnetic resonance unit (Philips Ingenia) with a 16‐element phased‐array head coil (Table 2). Resting‐state fMRI is used to examine intrinsic brain networks associated with consciousness by measuring spontaneous low‐frequency fluctuations in the blood oxygen level‐dependent signals. APT is a molecular MRI technique based on chemical exchange saturation transfer that measures the amide proton content, indirectly reflecting the protein content and pH value of a substance. IVIM uses multiple low b values to uncouple restricted diffusion from the pseudo‐diffusion effect of water movement in the capillary bed and can be used to non‐invasively quantify microperfusion. NODDI is an advanced non‐Gaussian diffusion model that can quantitatively estimate specific microstructural changes in terms of neurite density and orientation distribution of axons and dendrites.

**TABLE 2:** Behavioral, neurophysiological, and neuroimaging evaluations.

Items	Time consumed	Aim	Key parameters
Behavioral			
CRS‐R	20 min	Evaluate the neurobehavioral performance	Auditory, visual, motor, verbal, communication, arousal scale
BCI	20 min	Assess the severity of DOC	Accuracy
Neurophysiological			
EEG	24 h	Monitor electrical activity in the brain	Power, ABCD pattern, connectivity, sleep EEG pattern
SEP	30 min	Evaluate the integrity of the sensory pathway	Amplitude, latency
BAEP		Evaluate the integrity of the auditory pathways	Amplitude, latency
Neuroimaging			
rs‐fMRI	45 min	Examine spontaneous brain function by using blood oxygen level–dependent contrast	Amplitude of low‐frequency fluctuations, networks, connectivity
APT		Reflect the protein metabolism in specific brain areas	Magnetization transfer ratio asymmetry
IVIM		Reflect the microcirculation perfusion in specific brain areas	Perfusion fraction, molecular diffusion coefficient, pseudo‐diffusion coefficient
NODDI		Assess the microstructural complexity of neuronal axons and dendrites	Neurite density index, orientation dispersion index, isotropic volume fraction

Abbreviations: APT, amide proton transfer; BAEP, brainstem auditory evoked potential; BCI, brain–computer interface; CRS‐R, Coma Recovery Scale‐Revised; EEG, electroencephalogram; IVIM, intravoxel incoherent motion; NODDI, neurite orientation dispersion and density imaging; rs‐fMRI, resting‐state functional MRI; SEP, somatosensory evoked potential.

3D T1‐weighted images (170 transversal slices, repetition time = 2100 ms, voxel size = 1.0 × 1.0 × 1.0 mm^3^, flip angle = 8°, field of view = 250 × 199 × 170 mm^3^) are acquired before rs‐fMRI (EPI, gradient echo, volumes = 300, repetition time = 2000 ms, echo time = 30 ms, flip angle = 78°, voxel size = 3 × 3 × 3 mm^3^, field of view = 192 × 192 mm^2^, 32 transversal slices). DOC patients should be awake during the fMRI examination, and an arousal facilitation protocol (from the CRS‐R score) should be used if the patient exhibits sustained eyelid closure before the examination. Healthy subjects are instructed to keep their eyes open and to be in a relaxed state during the MRI data acquisition.

APT images are acquired using the 3D TSE‐Dixon approach with a continuous saturation duration of 2 s and an RF saturation amplitude of 2 µT. Saturation frequency offsets were implemented at 3.5, 3.5 ± 0.8, −3.5, −3.5 ± 0.8, and −1560 ppm. At 3.5 ppm, three Z‐spectral images are acquired using a different echo time shift on the order of 0.5 ms. This allows calculating a B0 field map directly based on mDIXON algorithms and then for z‐spectrum correction on a voxel‐by‐voxel basis. The far‐off resonant frequency (−1560 ppm) is acquired for signal normalization. Other imaging parameters were as follows: repetition time /echo time = 6306/7.8; field of view = 210 mm; slice thickness = 6 mm; sensitivity encoding factor = 2. Acquisition time is 11 min 27 s.

IVIM is performed with a short multiple *b* value diffusion‐weighted MRI sequence, using a monopolar pulsed gradient scheme and a spin‐echo‐planar imaging readout, with the following acquisition measures: 10 *b* values (0, 10, 20, 50, 100, 150, 200, 500, 800, 1000 s/mm^2^) in 3 orthogonal directions, repetition time = 7317 ms, echo time = 83 ms, field of view = 210 × 210 mm^2^, slice thickness = 2 mm, acquisition matrix = 104 × 102, in‐plane physical resolution = 2.0 ×2.0 mm^2^, parallel imaging accelerating factor 2.1, 65.3% Fourier encoding. Acquisition time is 4 min and 45 s.

For the NODDI diffusion scan, the field of view is 232 mm in *X* and *Y* and 162 mm in the *Z* direction, with 2.0 mm isotropic voxels. The echo and repetition times were 105 and 5076 ms respectively. Data consist of 127 volumes with 13 non‐diffusion‐weighted images (*b* = 0 s/mm^2^), and 114 diffusion‐encoding gradient directions (6*b* = 500, 48*b* = 1000, and 60*b* = 2000 s/mm^2^), evenly spread over the entire spherical shells using an electrostatic repulsion model, and interspersed in time to minimize gradient heating. Acquisition time is 10 min and 56 s.

### 2.12 Sample Size

No prior studies have examined the efficacy of EA in patients with DOC. Therefore, the sample size calculation was based on a clinical trial of transcranial direct current stimulation (tDCS) evaluating its effect on consciousness recovery in DOC patients (Barra et al. 2022). In that study, the total change in the CRS‐R score after sham treatment was −0.1 (±1.6) from baseline, while the total change after tDCS treatment was 1 (±2.6). For this study, we assumed that the sham‐EA group would show a total change in the CRS‐R score of 0 (±1.6), and that EA treatment would result in a 1.3‐point improvement in the CRS‐R score. Using a two‐sided significance test with a 5% type I error, we calculated a minimum of 24 participants per group. To account for a 4% non‐adherence to the treatment protocol and loss to follow‐up, the sample size is set at 50 (25 per group). There is no limitation on the sample size for subgroup stratification.

### 2.13 Statistical and Analytical Plan

Under the Intention To Treat principle, all patients who are randomized are included in the analysis of the original allocation group. Baseline and demographic characteristics such as gender, age, time from injury, brain injury location, closed or open TBI, right‐ or left‐handed, GCS score, CRS‐R score, APACHE2 score, etc., will be pooled by treatment group to assess comparability of treatment groups. The primary outcome of the change in CRS‐R score after 14‐day treatment, secondary outcomes of BCI accuracy after 14‐day treatment and the GOS‐E score at 30 days and 90 days will be analyzed using Student's t‐test or Mann‐Whitney U test (whichever is proper) and will be analyzed by bootstrap analyses in general linear models to estimate adjusted odds ratios and associated 95% confidential intervals (adjusted for age, sex, time from injury to enrollment, and baseline CRS score). Two‐sided p values ≤ 0.05 will be considered significant. The pairwise deletion method is used to handle missing data. Statistical analysis will be performed with SPSS version 22 software (SPSS Inc., Chicago, IL, USA).

EEG data are analyzed using MATLAB 2016b (Mathworks, Sherborn, Massachusetts, USA). The immediate effects of EA are explored by analyzing the mean EEG power, pattern, and connectivity at 10 min before the allocated treatment and at four time points after the allocated treatment (10 min, 60 min, 6 h, and 24 h). The prolonged effects of EA are explored by analyzing changes in EEG power, pattern, and connectivity before and after the 14‐day intervention. For each electrode, the mean power values of 5 s epochs are estimated in different standard EEG frequency bands: delta (0.5–4 Hz), theta (4–8 Hz), alpha (8–13 Hz), and beta (13–30 Hz). EEG patterns are determined based on the EEG power spectrum and classified into four categories according to the “ABCD” model, which decodes the integrity of thalamocortical circuitry and the preservation of consciousness (Alkhachroum et al. 2020; Edlow et al. 2021). Functional connectivity is estimated using phase‐synchronization measures. Sleep EEG is rated using the modified Valente's Grade (Yang et al. 2020). For SEP data, the average of N20–P25 peak‐peak amplitude from the right and left sides is calculated. For BAEP, wave IV–V peak‐to‐peak amplitude, wave V latency in ms, and interpeak latency difference (IPL) III–V are selected as main variables.

The preprocessing of fMRI data is conducted using MELODIC in the FMRIB Software Library. The preprocessing pipeline includes removal of the first five volumes to allow signal stabilization, motion correction using MCFLIRT, brain extraction via BET, spatial smoothing with a 5 mm full‐width‐at‐half‐maximum Gaussian kernel, rigid‐body registration, high‐pass filtering with a cutoff of 100 s to remove low‐frequency drifts, and single‐session independent component analysis with automatic dimensionality estimation, and removal of noise components and lesion‐driven artifacts using FIX. The changes in the amplitude of low‐frequency fluctuations of the region of interest are analyzed. Seed‐based correlation analysis is performed with DPARSF‐A using a 6 mm radius sphere for each node of the seven networks: higher‐order networks (the default mode network, dorsal attention network, executive control network, and salience network) and sensory‐related lower‐order networks including the visual network, sensory input auditory network, and sensorimotor network. Group‐level analyses are performed using Statistical NonParametric Mapping (http://www.nisox.org/Software/SnPM13/) for each network. The APT imaging analysis is performed using the IntelliSpace Portal v.8.0.1 (Philips Healthcare). The quantitative APT analysis is performed in selected ROIs, which include cortex (frontal, parietal, temporal cortex, occipital), periventricular white matter, thalamus, basal ganglia, insular lobe, hippocampus, parahippocampal gyrus, corpus callosum, cerebellum, brainstem (midbrain, pons, medulla). The variability in the size of each ROI across subjects is less than 5 mm^2^. The IVIM biexponential signal equation model is fitted using a homemade C++ implementation of the standard 2‐step algorithms based on the Levenberg‐Marquardt method. IVIM f is defined as perfusion fraction, D* is defined as pseudo‐diffusion coefficient related to microcapillary perfusion, fD* is defined as the multiplication of *f* and D* (related to blood flow), and D is defined as molecular diffusion coefficient. The preprocessing of NODDI data includes denoising using random matrix theory, correction for head motion and eddy current distortion, Gibbs‐ringing artifact removal, and Rician debiasing. NODDI biophysical modeling method divides water diffusion in the brain into three microstructural compartments: intracellular space through the Neurite Density Index, Orientation Dispersion Index, and Isotropic Volume Fraction. Diffusion tensors are fitted for both the multi‐shell and extracted *b* = 1000 data using a non‐linear least‐squares fitting algorithm implemented in dipy (Garyfallidis et al. 2014), from which fractional anisotropy and mean diffusivity images are generated. The NODDI model is fit by the Accelerated Microstructure Imaging via Convex Optimization implementation in Python (Daducci et al. 2015). Multiple comparisons are corrected using False Discovery Rate.

### 2.14 Quality Control

This study will be conducted by the guidelines of the International Conference on Harmonisation of Good Clinical Practice, and all relevant national and local regulations. The Quality Control and Assurance Committee (QCAC) is responsible for the assessment of clinical care, serious adverse events (SAE) review, and adjudication for reported relatedness of an SAE to treatment and the duration of the trial (if necessary). The protocol will be amended, or the study will be stopped earlier if an excess of particular SAEs appears to be protocol‐related. The management of SAEs includes no action taken, suspension or termination of the allocated intervention, administration of concomitant drugs, inpatient hospitalization, or prolongation of existing hospitalization. The QCAC members are not directly involved with the trial.

### 2.15 Confidentiality

All study investigators have the responsibility to protect all personal identity and medical information at all times. All the medical records of study participants, such as consent, case report forms, and reports of tests and examinations, will be securely stored. Only de‐identified data will be submitted to the Statistical Analysis Center. The trial sponsor, PI, and ethics committee are allowed to refer to medical records, in the course of monitoring data quality and adherence to the study protocol.

### 2.16 Dissemination

The results of this trial will be disseminated to a wide clinical audience (patients, health professionals, policymakers, and the general public) through publication in a high‐impact international scientific journal.

## 3 Discussion

DOC is a common consequence of TBI, but the clinical management of DOC remains a great challenge despite many efforts devoted to this long‐standing problem (Thibaut et al. 2019). Many ancient records and modern studies have shown that acupuncture may have a beneficial effect on DOC due to TBI (Cavalli et al. 2018). The AcuDoc trial is the first randomized and sham‐controlled trial to investigate the therapeutic effects and mechanisms of EA in TBI‐induced DOC through a multimodal approach.

Previous randomized trials of non‐pharmacological interventions for DOC have sample sizes ranging from 10 to 30^3^ (Barra et al. 2022; Spaccavento et al. 2024; Bai et al. 2017; Angelakis et al. 2014). In this trial, we plan to enroll 50 patients, and this sample size can detect a clinically important difference of 1.3 in the total score change of CRS‐R between the two groups. Moreover, previous trials usually include DOC patients with all kinds of etiologies (Hu et al. 2024), such as stroke, hypoxic‐ischemic encephalopathy, and TBI. However, outcomes vary widely by etiology, which will bias the assessment of treatment efficacy. This trial only included patients with DOC due to TBI within 3 months and used stratified randomization to ensure balanced proportions of UWS and MCS in the two groups to minimize biases. Blinding is a challenging yet crucial procedure in conducting randomized trials of non‐pharmacological therapies for DOC (Martens et al. 2020). However, very few non‐pharmacological DOC studies have employed a sham control, and none of the previous trials investigating the effects of acupuncture or EA on DOC or coma were sham‐controlled (Chen et al. 2021; Xin et al. 2022; Matsumoto‐Miyazaki et al. 2016). Although many DOC patients are unable to perceive whether or not they are receiving acupuncture treatments, the medical care provided by physicians, nurses, or caregivers may be biased by the setting of unblinding. In this trial, we adopted a classical masking method used in previous high‐quality randomized trials of EA (Liu et al. 2017): sham acupoints, sham acupuncture, and sham conductor wire are used in the sham‐controlled group.

Accurately assessing the degree of impaired consciousness has been a clinical challenge. Behavioral assessments (e.g., CRS‐R) are the primary method of grading DOC. However, some DOC patients may retain sentience and intentional thought, but due to impaired motor pathways, they are unable to use their behaviors to complete commanded tasks, i.e., in the cognitive‐moto dissociation state, which leads to bias and misdiagnosis when relying on behavioral scales alone to assess the level of consciousness. BCI technology can create a direct communication channel between the brain and the external environment without passing through peripheral nerves, bones, or muscles, providing a new way of revealing the covert consciousness that exists in some DOC patients. In this trial, we use a previously published BCI consciousness assessment system (Pan et al. 2020) as a complement to the CRS‐R. This BCI system analyzes the P300 and SSVEPs generated in the patient's brain when commands are executed. It determines whether the patient can gaze at the instructed photos according to the commands and simultaneously provides feedback on classification results, thus assessing whether the patient can comprehend and complete the commands, i.e., whether the patient has covert consciousness (Pan et al. 2020). This trial is the first attempt to use the BCI technique to assess treatment outcomes in patients with DOC, thus complementing some of the deficiencies of behavioral scales and providing a more accurate benchmark for exploring the therapeutic mechanisms of EA.

Multiple neurophysiological and neuroimaging techniques are used in this trial to explore potential therapeutic mechanisms of EA. EEG can dynamically record the real‐time changes in brain function for a sustained long period at a relatively low cost and provide a large amount of EEG data for quantitative analysis (Edlow et al. 2021; Wutzl et al. 2021; Curley et al. 2018). Previous studies have shown that the powers of the delta and alpha bands are correlated with CRS‐R scores, i.e., alpha power increases and delta power decreases with increasing CRS‐R values, UWS patients had decreased alpha power but increased delta power, compared with MCS patients (Lehembre et al. 2012; Lechinger et al. 2013; Chennu et al. 2014). The “ABCD” model is hypothesized to reflect the severity of structural or functional deafferentation by organizing and categorizing sequential changes in EEG power spectra (Edlow et al. 2021). EEG connectivity estimates the severity of large‐scale brain networks, and its measures are significantly correlated with the level of DOC (Chennu et al. 2014; Naro et al. 2018). With a higher spatial resolution and better integration with structural lesions, rs‐fMRI reveals functional connectivity within brain networks by measuring the blood‐oxygen‐level‐dependent signal (Bodien et al. 2019; Snider and Edlow 2020). Multiple resting‐state networks, such as the default mode, frontoparietal, salience, auditory, sensorimotor, and visual networks, reflect intrinsic brain activities and have been used to discriminate UWS patients from MCS patients with a high discriminative capacity (Gosseries et al. 2016; Vanhaudenhuyse et al. 2010; Demertzi et al. 2015). APT imaging is a novel molecular imaging technique that reflects the concentration of mobile macromolecules and utilizes amide protons in endogenous mobile proteins and peptides in tissues to generate images, which in turn present metabolic conditions and physiopathological information inside living cells (Msayib et al. 2019; Chen et al. 2023). APT imaging can be used to explore the protein metabolism in key brain regions of DOC patients at the molecular level. IVIM imaging is an advanced diffusion technique derived based on diffusion‐weighted imaging, which can distinguish pure water molecule diffusion from microvascular perfusion diffusion, and can obtain the true diffusion coefficient (*D*) value, which represents the true water molecule diffusion, and the pseudo‐diffusion coefficient (D*) value, which represents perfusion‐related diffusion (Bergamino et al. 2022; van Dinther et al. 2022). IVIM imaging can reflect the specific brain tissue blood microcirculation perfusion by measuring the degree of molecular diffusion within the voxel, and assess the damage and repair of the brain regions related to consciousness from the perspective of microperfusion. NODDI is an emerging magnetic resonance diffusion imaging, which can be used to assess the microstructural complexity of neuronal axons and dendrites, thus reflecting information on the morphology of nerve fibers, allowing more direct and specific measurements of tissue microstructure (Chen et al. 2021; Huang et al. 2023). The NODDI quantifies the dispersion and density of neural axons and quantitatively estimates the dendritic distribution of dendrites, which can directly reflect the complexity of grey matter and can be used to assess the degree of injury to the conscious‐related structures of DOC patients. Through these neurophysiological and neuroimaging techniques, the therapeutic mechanisms of EA in treating DOC can be explored from multiple perspectives, including electrical activity, metabolism, microperfusion, neural axon injury in brain areas critical for the restoration of consciousness, structural and functional integrity of consciousness‐related neural circuits, and interactions between key brain regions and the organization of resting‐state networks.

DOC remains a complex clinical challenge that requires multifaceted treatment approaches, necessitating further exploration of therapeutic options. Historical medical records and contemporary research suggest that acupuncture may hold potential for facilitating the recovery of consciousness. Additionally, acupuncture is characterized by its safety, accessibility, and compatibility with other pharmacological and non‐pharmacological treatments. This study will be the first randomized sham‐controlled trial to investigate the clinical benefits of EA in patients with DOC. Through rigorous design and multimodal evaluation, this study aims to provide Class II evidence regarding the efficacy of EA in treating DOC and to explore the underlying therapeutic mechanisms. Some limitations of this study should be pointed out. First, it is a single‐center trial with a limited sample size, which may generate some selection bias. The subgroup analyses are further constrained by even smaller numbers, potentially limiting their statistical power. Second, the EA treatment course in this study is set at two weeks, whereas in clinical practice, DOC therapeutic interventions typically require a much longer duration. The effects of EA on the GOS‐E score at 30 and 90 days should be interpreted with caution. Should this study demonstrate promising effects of EA, the potential for sequential EA therapy could be explored in future multicenter studies. Third, patients who develop DOC within 7–28 days of onset are also included, and they are not the typical population of prolonged DOC. We noticed that by the time the patient evolves from coma to UWS or MCS, his or her brain injury usually has become stable. Many patients with TBI evolve to DOC earlier than 28 days (e.g., 14 or 20 days), and these patients may benefit most from the early intervention for DOC. From an ethical and practical perspective, physicians usually choose to initiate therapies early to improve consciousness in patients with DOC and rarely delay treatment because it is not yet 28 days. Some trials also focused on this period of DOC (Vitello et al. 2023). Fourth, rehabilitation interventions are not restricted in this trial, so we cannot rule out a synergistic effect. The rehabilitation plans are carried out by the same team of physicians in this study and are the same for both groups. In clinical practice, interventions for conscious recovery and physical rehabilitation are commonly used together for patients with DOC. Fifth, estimating the sample size based on tDCS research data may introduce bias. Moreover, while the effects of EA are evaluated from multiple perspectives in this study, the use of multiple secondary outcomes may increase the risk of false positives.

In conclusion, we designed a randomized, participant‐blinded, sham‐EA‐controlled trial to explore the therapeutic effects of EA in DOC due to TBI by means of behavioral and BCI assessments as well as neurophysiological and neuroimaging techniques. Our trial will bring evidence for the benefits and potential therapeutic mechanisms of EA in the treatment of DOC and provide a scientific basis for conducting a multicenter study with a large sample size in the future.

## Author Contributions

**Kaiqi Lin**: investigation, writing – review and editing, writing – original draft, formal analysis. **Jixiang Chen**: investigation, writing – review and editing, formal analysis. **Jiahui Pan**: software, writing – review and editing, project administration, formal analysis, investigation. **Ruihong Wang**: investigation, writing – review and editing, formal analysis. **Shibiao Wu**: investigation, writing – review and editing, formal analysis. **Wanxing Wen**: investigation, writing – review and editing, formal analysis. **Yuanqing Li**: conceptualization, methodology, investigation, writing – review and editing, resources, formal analysis, supervision. **Lixin Wang**: supervision, project administration, conceptualization, methodology, writing – review and editing. **Fang Yuan**: project administration, supervision, conceptualization, methodology, writing – review and editing, writing – original draft, funding acquisition, resources.

## Conflicts of Interest

The authors declare no conflicts of interest.

## Peer Review

The peer review history for this article is available at https://publons.com/publon/10.1002/brb3.70637

## Supporting information




**Supporting Checklist**: brb370637‐sup‐0001‐Checklist.doc

## Data Availability

All data are available in the main text.
